# The zinc-ribbon domain of *Helicobacter pylori *HP0958: requirement for RpoN accumulation and possible roles of homologs in other bacteria

**DOI:** 10.1186/2042-5783-1-8

**Published:** 2011-08-23

**Authors:** Lara E Pereira, Jennifer Tsang, Jan Mrázek, Timothy R Hoover

**Affiliations:** 1 Emory Vaccine Center, 954 Gatewood Road, Emory University, Atlanta, GA 30329, USA; 2 Department of Microbiology, University of Georgia, Athens, GA 30602, USA; 3 Institute of Bioinformatics, University of Georgia, Athens, GA 30602, USA

**Keywords:** Helicobacter pylori, sigma54, RpoN, HP0958, FlgZ

## Abstract

**Background:**

*Helicobacter pylori *HP0958 protein (FlgZ) prevents the rapid turnover of RpoN (σ^54^), a transcription factor required for expression of several flagellar genes in *H. pylori*. FlgZ possesses a zinc-ribbon domain (DUF164) that contains two conserved CXXC motifs which coordinate a zinc ion and is thought to interact with nucleic acids or proteins. Two conserved cysteine residues in FlgZ (Cys-202 and Cys-223) were replaced with serine to assess their significance in FlgZ function. After confirming the importance of the CXXC motifs in the DUF164 domain of FlgZ, the distribution of DUF164 proteins and RpoN homologs in other bacteria was examined to determine if a correlation existed for the concurrence of the two proteins.

**Results:**

Levels of RpoN were greatly reduced in *H. pylori *strains that expressed the FlgZ^C202S ^or FlgZ^C223S ^variants. The FlgZ^C202S ^variant, but not the FlgZ^C223S ^variant, accumulated at levels similar to the wild-type protein. DUF164 proteins are not universally distributed and appear to be absent in several major bacterial taxa, including Cyanobacteria as well as Alpha-, Beta- and Gammaproteobacteria. With the exception of the Actinobacteria, members of which generally lack RpoN, genes encoding DUF164 proteins and RpoN are frequently found in the same genome. Interestingly, many of the DUF164 proteins in Actinobacteria and Bacteroidetes lack most or even all of the conserved cysteine residues.

**Conclusions:**

These findings suggest the importance of the zinc-ribbon domain of FlgZ in protecting RpoN from turnover. Since many bacteria that possess a DUF164 protein also contain RpoN, DUF164 proteins may have roles in RpoN protection or function in other bacteria.

## Background

*Helicobacter pylori *is a member of the Epsilonproteobacteria that colonizes the human gastric mucosa where it causes a variety of gastrointestinal diseases, including acute gastritis, peptic and duodenal ulcers, B cell MALT lymphoma, and gastric adenocarcinoma [1-3]. Colonization of the gastric mucosa by *H. pylori *requires the bacterium to be motile [4,5], which is achieved through a cluster of polar sheathed flagella.

The bacterial flagellum is a complex structure consisting of three basic substructures - the basal body, hook and filament. Dozens of genes are required for flagellar biogenesis and the expression of these genes is regulated by a transcriptional hierarchy in which genes are expressed as their products are needed for assembly [6,7]. All three of the RNA polymerase sigma (σ) factors in *H. pylori *are involved in flagellar biogenesis. In general, genes needed early in flagellar assembly are under control of the primary σ factor RpoD (σ^80^), while RpoN (σ^54^) is responsible for transcription of genes needed midway through flagellar assembly and transcription of late flagellar genes is dependent on FliA (σ^28^) [8-13].

Transcriptional activation of the RpoN-dependent flagellar genes in *H. pylori *requires the response regulator FlgR and its cognate histidine kinase FlgS [12,14]. Although the signaling pathway that regulates expression of the RpoN flagellar regulon is poorly understood, transcriptional control of the regulon has been shown to be intimately associated with the flagellar protein export apparatus in *H. pylori *and its close relative *Campylobacter jejuni *[10,15-18]. Transcription of the *H. pylori *RpoN regulon also requires the putative RpoN chaperone and zinc-ribbon domain protein HP0958 [19,20], which we hereafter refer to as FlgZ (flagellar-associated zinc-ribbon domain protein).

FlgZ was originally identified as interacting with RpoN in a high-throughput screen of a yeast two-hybrid system [21]. Subsequently, FlgZ was shown to be required for motility in *H. pylori *[19,20] and to prevent the rapid turnover of RpoN - the half-life of RpoN is > 4 hours in wild type compared to ~30 minutes in the *flgZ *mutant [19]. FlgZ may have additional roles in *H. pylori *motility as it also interacts with the flagellar protein export apparatus protein FliH in the yeast two-hybrid system [21]. In addition, Douillard and co-workers showed that FlgZ binds *flaA *transcript (*flaA *encodes the major flagellin of *H. pylori*) and is needed for optimal production of FlaA [22]. These researchers proposed that FlgZ may work together with FliH to direct *flaA *transcripts to the basal body of the nascent flagellum to couple the translation and secretion of FlaA [22]. Although FlgZ may play a direct role in the expression and export of FlaA, this activity is not essential for motility since overproduction of RpoN suppresses the motility defect of the *flgZ *mutation [19].

FlgZ is a 254 amino acid polypeptide and a recently solved crystal structure of the protein revealed that it consists of a highly elongated, kinked coiled-coil hairpin domain (resides 1-170) followed by a zinc-ribbon domain (residues 174-238) [23]. FlgZ is a member of a family of proteins which contain a predicted zinc-ribbon domain of unknown function (PF02591; COG1579). Most members of this protein family, including FlgZ, contain the motif Y/F_X_18-23__ CXXC_X_18-26__CXXC, in which the conserved cysteine residues coordinate a zinc ion [23]. Zinc fingers function as interaction modules that bind DNA, RNA, proteins or small molecules [24-28]. Given the potential for FlgZ to act as both an RpoN chaperone and a RNA chaperone in a coupled translation-protein secretion process [19,22], it is unclear which of these functions is associated with the FlgZ zinc-ribbon domain.

To examine the issue of whether the zinc-ribbon domain of FlgZ is needed to protect RpoN from turnover in *H. pylori*, we changed two of the conserved cysteine residues in FlgZ (Cys-202 and Cys-223) to serine residues and examined the phenotypes of *H. pylori *strains expressing the FlgZ variants. Strains expressing either the FlgZ^C202S ^or FlgZ^C223S ^variant were non-motile and had reduced levels of RpoN, indicating the importance of these conserved cysteine residues in the function or stability of FlgZ. A search of bacterial genome sequences revealed that homologs of FlgZ (characterized by the presence of the DUF164) are widespread among bacterial species, but appear to be absent in several major taxonomic groups. In many taxonomic groups there is a statistically significant correlation between the presence of FlgZ homologs and RpoN, suggesting a role for FlgZ homologs in RpoN function in a diverse set of bacteria.

## Results and discussion

### Conserved cysteine residues in the zinc-ribbon domain of FlgZ are important for function and stability

**Figure 1:**
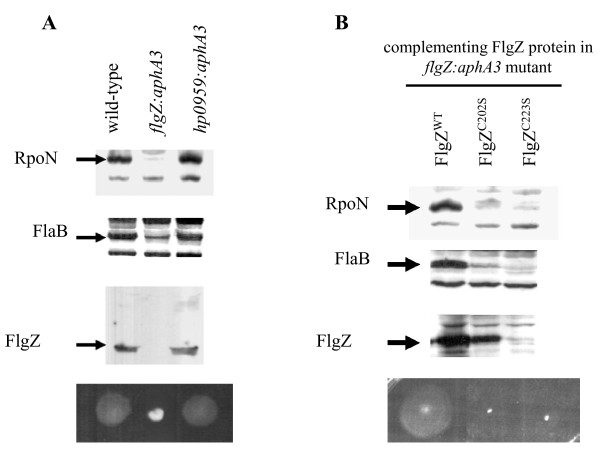
**Effects of FlgZ variants on motility and RpoN stability**. (**A**) Wild-type *H. pylori *strain ATCC 43504, *flgZ*:*aphA3 *and *hp0959*:*aphA3 *mutants were analyzed by western blotting for the proteins indicated in the top three panels. Construction of the mutant strains was described previously [19]. Approximately 1 × 10^8 ^cells were lysed and loaded in each lane. Following protein transfer, nitrocellulose membranes were probed with antiserum directed against RpoN, FlaB or FlgZ proteins that were fused to the maltose-binding protein (MBP) as described [19]. Results of motility assays are shown in the bottom panel. Each strain was inoculated into semisolid motility agar plates using a sterile toothpick and incubated 5 days at 37^®^C under microaerophilic conditions as described previously [19]. (**B**) Wild-type or mutant alleles of *flgZ *were introduced into the *hp0405 *locus of the *H. pylori flgZ*:*aphA3 *mutant as described previously [19]. The resulting strains were analyzed by western blotting for RpoN, FlaB and FlgZ (top three panels) and for motility (bottom panel) as described for panel A.

To determine if the conserved cysteine residues in the zinc-ribbon domain of FlgZ are critical for the function of the protein, mutations were introduced in each of the conserved CXXC motifs and the phenotypes of strains expressing the resulting FlgZ variants were examined. The conserved cysteine residues in *H. pylori *26695 FlgZ are Cys-199, Cys-202, Cys-223 and Cys-226. Cys-202 and Cys-223 were changed to serine residues and the resulting FlgZ variants were expressed in a mutant strain of *H. pylori *43504 in which *flgZ *(*hp0958*) was disrupted with a kanamycin-resistance cassette. As shown in Figure 1A and reported previously, disruption of *flgZ *in *H. pylori *resulted in a loss of motility and reduced RpoN levels [19]. Wild-type FlgZ expressed from the *hp0405 *locus in the *flgZ *mutant restored motility and supported wild-type levels of RpoN and FlaB (Figure 1B). FlaB is a minor flagellin whose expression is dependent on RpoN [10,12,29], and levels of this flagellin were reduced in the *flgZ *mutant. In contrast to wild-type FlgZ, the FlgZ^C202S ^and FlgZ^C223S ^variants failed to restore motility or support wild-type levels of RpoN and FlaB. Western blot analysis showed that the FlgZ^C202S ^variant was expressed at near wild-type levels in *H. pylori*. Taken together, these data suggest that the zinc-ribbon domain of FlgZ has a role in protecting RpoN from turnover. It is unclear if the FlgZ^C202S ^variant is defective in binding RpoN or in a subsequent step needed to protect RpoN from turnover.

In contrast to the FlgZ^C202S ^variant, the FlgZ^C223S ^variant was not expressed stably in *H. pylori*. DNA sequencing of the *flgZ *allele encoding the FlgZ^C223S ^variant failed to reveal any additional mutations, suggesting that the serine substitution at Cys-223 decreases the stability of the protein. Caly and co-workers [23] reported that changing Cys-199 of FlgZ to alanine did not significantly alter the structure of the protein as assessed by circular dichroism. Taken together, these observations suggest substitutions in the second CXXC motif have a more profound effect on the structure of FlgZ than those in the first CXXC motif, while substitutions in either of the two CXXC motifs result in reduced RpoN levels and loss of motility.

### Taxonomic distribution of proteins containing the DUF164

Having demonstrated the importance of at least one of the conserved cysteine residues within the DUF164 of FlgZ in protecting RpoN from turnover in *H. pylori*, we wished to determine if DUF164 proteins in other bacteria could have a role in RpoN function similar to that of FlgZ. To address this issue we investigated if the presence of a FlgZ homolog in a genome correlated with that of RpoN. For this purpose, we defined FlgZ homologs as proteins containing the DUF164 domain (PF02519), whereas all proteins belonging to the COG1508 according to the IMG annotations were considered RpoN homologs. We performed our analysis with a dataset restricted to finished genome sequences as well as the complete collection of both finished and draft sequences with the caveat that the apparent absence of either gene in a draft genome could be due to gaps in sequence coverage. The results of the analysis, which are summarized in Table 1 revealed several intriguing trends.

**Table 1:** Taxonomic distribution of DUF164 proteins and RpoN in the domain Bacteria

Taxonomic group	^a^Number of genomes with both DUF164 protein and RpoN	^a^Number of genomes with DUF164 protein only	^a^Number of genomes with RpoN only	^a^Number of genomes with neither DUF164 protein nor RpoN
Acidobacteria	2 (2)	0 (0)	1 (1)	0 (0)

Actinobacteria	1 (2)	73 (143)	0 (0)	16 (37)

Aquificae	6 (8)	0 (0)	0 (0)	0 (0)

Bacteroidetes	24 (80)	0 (2)	0 (0)	2 (4)

Chlamydiae	15 (22)	0 (0)	0 (0)	0 (0)

Chlorobi	11 (12)	0 (0)	0 (0)	0 (0)

Chloroflexi	1 (2)	3 (3)	3 (5)	6 (6)

Cyanobacteria	0 (0)	0 (0)	0 (0)	38 (51)

Deferribacteres	0 (1)	0 (0)	0 (0)	0 (0)

Dictyoglomi	0 (0)	2 (2)	0 (0)	0 (0)

Elusimicrobia	0 (0)	1 (1)	0 (0)	1 (1)

Fibrobacteres	0 (0)	0 (0)	1 (2)	0 (0)

Firmicutes	17 (23)	0 (1)	76 (240)	100 (243)
Clostridia	17 (23)	0 (1)	25 (71)	11 (55)
*Clostridium*	12 (16)	0 (1)	10 (39)	3 (12)

Fusobacteria	0 (0)	0 (0)	0 (12)	4 (8)

Gemmatimonadetes	1 (1)	0 (0)	0 (0)	0 (0)

Lentisphaerae	2 (2)	0 (0)	0 (0)	0 (0)

Nitrospirae	1 (1)	0 (0)	0 (0)	0 (0)

Planctomycetes	3 (7)	0 (0)	0 (0)	0 (0)

Alphaproteobacteria	0 (0)	0 (0)	79 (122)	42 (75)

Betaproteobacteria	0 (0)	0 (0)	69 (141)	3 (4)

Deltaproteobacteria	32 (41)	0 (1)	1 (1)	0 (0)

Epsilonproteobacteria	22 (43)	4 (5)	0 (2)	0 (1)

Gammaproteobacteria	0 (0)	0 (0)	199 (368)	44 (71)

Magnetococci	0 (0)	0 (0)	1 (1)	0 (0)

Zetaproteobacteria	0 (0)	0 (0)	0 (1)	0 (0)

Spirochaetes	10 (27)	2 (3)	6 (6)	0 (0)

Synergistetes	0 (0)	0 (0)	1 (3)	0 (1)

Tenericutes	0 (0)	0 (0)	0 (0)	25 (40)

Thermi	0 (0)	5 (8)	0 (0)	0 (0)

Thermotogae	0 (0)	0 (0)	3 (3)	8 (9)

TM7	0 (0)	0 (0)	0 (0)	0 (1)

Verrucomicrobia	3 (6)	0 (0)	0 (1)	0 (0)

WWE1	1 (1)	0 (0)	0 (0)	0 (0)

Total	150 (281)	90 (169)	440 (909)	289 (552)

^a^Numbers outside parentheses indicate results from analysis of finished genome sequence, while numbers within parentheses indicate results for both finished and unfinished genome sequences.

First, proteins characterized by the presence of the DUF164 are essentially restricted to the domain Bacteria as there are no representatives in the domain Archaea and only one in the domain Eukaryota. The sole eukaryotic protein is present in the sac fungus *Phaeosphaeria nodorum *SN15, and it differs substantially from FlgZ in both its size (571 amino acids in length versus 254 amino acids) and the presence of a SET domain (PF00856). Second, several bacterial taxonomic groups that are well represented in the JGI IMG database, such as Cyanobacteria, Alphaproteobacteria, Betaproteobacteria, Gammaproteobacteria, and Tenericutes, lack proteins that contain DUF164 (Table 1). Third, with only one exception, bacteria do not possess more than one DUF164 protein. The only exception we found in the database was *Opitutus terrae *PB90-1 (phylum Verrucomicrobia) which has two DUF164 proteins.

As shown in Table 1 DUF164 proteins are found in a broad range of taxonomic groups. While members of many of these groups, including Bacteroidetes, Chlamydiae, Chlorobi, Planctomycetes, Deltaproteobacteria, Epsilonproteobacteria, Spirochaetes, and Verrucomicrobia, often possess RpoN, members of Actinobacteria and Thermi typically lack RpoN. Indeed, homologs of *rpoN *are present in only two of 182 Actinobacteria genomes and none of the eight Thermi genomes in the JGI IMG database, indicating that the DUF164 proteins in these bacteria are obviously not involved in RpoN function.

We used the Fisher exact test to determine if there was a statistically significant association between DUF164 proteins and RpoN. The data from Table 1 were combined in a single 2-by-2 contingency table where the lines and columns contained the counts of genomes that possess and lack DUF164 proteins and RpoN, respectively. The online probability calculator from Vassar College (available at http://faculty.vassar.edu/lowry/VassarStats.html) was used to calculate the probabilities. One-tail probabilities obtained from the Fisher exact test close to zero (p < 0.05) indicate significant tendency of DUF164 and RpoN proteins to occur in the same genomes whereas probabilities close to 1 indicate a significant mutual avoidance of the two proteins. Analysis of the total finished genome sequences failed to reveal a significant tendency of DUF164 and RpoN to occur in the same genomes (p = 0.30). Analysis of a data set that included all of the finished genomes except the Actinobacteria genomes, however, indicated a significant tendency of DUF164 and RpoN proteins to occur in the same genomes (p < 10^-13^). A statistically significant association between DUF164 and RpoN proteins was also noted within the phylum Firmicutes (p < 10^-5^), where DUF164 proteins appear to be restricted to members of the class Clostridia (Table 1). The strong correlation between the presence of RpoN and DUF164 proteins in many bacterial genomes raises the possibility that, as with FlgZ, DUF164 proteins in these bacteria have a role in RpoN function.

Bacteria in taxonomic groups whose members generally possess both DUF164 and RpoN proteins often employ RpoN in unconventional ways. For example, *H. pylori *FlgR differs from most activators of σ^54^-RNA polymerase holoenzyme (σ^54^-holoenzyme) in that it lacks an enhancer-binding (i.e., DNA-binding) domain and activates transcription independently of an enhancer [29]. Many members of the Epsilonproteobacteria, Chlamydiae and Bacteroidetes groups similarly possess RpoN-dependent activators that lack enhancer-binding domains and presumably function as enhancer-independent activators [30-32]. Moreover, the RpoN-dependent activators *Campylobacter jejuni *FlgR (subphylum Epsilonproteobacteria) and *Borrelia burgdorferi *Rrp2 (phylum Spirochaetes) have been shown to activate transcription in an enhancer-independent manner [31,33]. Based on these comparisons, it is intriguing to speculate that FlgZ homologs may stimulate enhancer-independent transcriptional activation (which is presumably less efficient than enhancer-dependent transcription) in these bacteria. For instance, FlgZ homologs could facilitate interactions between RpoN and core RNA polymerase to increase the concentration of σ^54^-holoenzyme in the cell, resulting in increased promoter occupancy and allow for greater opportunity for productive interactions between the activator and σ^54^-holoenzyme.

Along a similar line of reasoning, competition among sigma factors for core RNA polymerase is an important factor in gene regulation [34]. If FlgZ and its homologs function as RpoN chaperones, such activity may allow RpoN to compete effectively with other sigma factors for binding RNA polymerase. This could be important for RpoN function in members of the Deltaproteobacteria which typically have exceptional numbers of RpoN-dependent activators (*Myxococcus xanthus *has ~50 different activators) and where RpoN is a significant global regulator in these bacteria [35,36].

### Architectural features of DUF164 proteins

**Figure 2:**
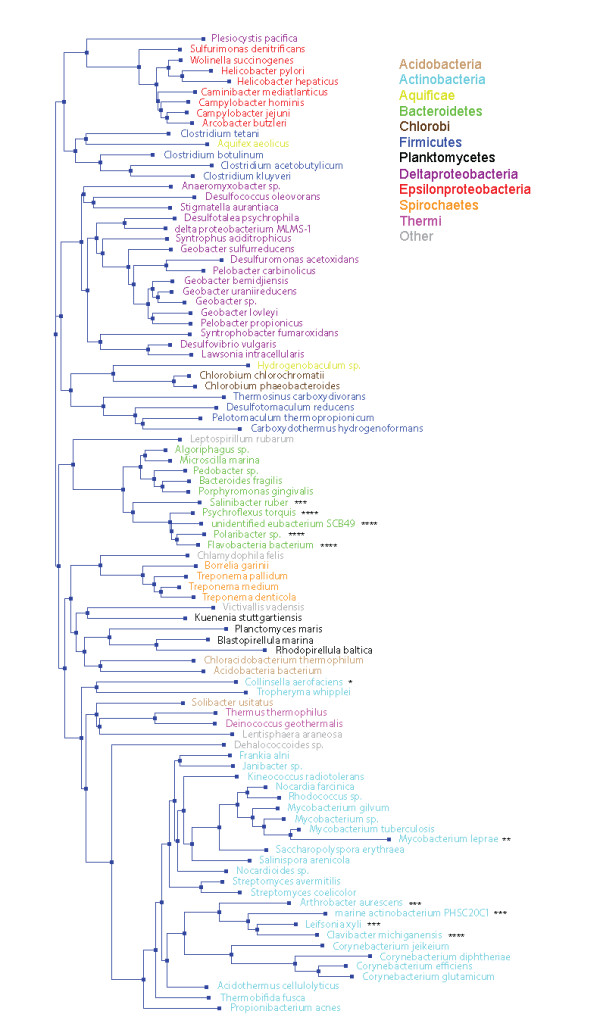
**Phylogentic tree for DUF164 domain proteins**. The figure presents a modified phylogenetic tree downloaded from Pfam (http://pfam.sanger.ac.uk/family?acc=PF02591). The tree was generated from an alignment of 93 seed DUF164 sequences using the FastTree algorithm [42]. Taxonomic groups for the bacterial species are color-coded. The numbers of stars indicate the number of cysteine residues in the conserved CXXC motifs that are replaced with other amino acid residues.

Divergence of DUF164 in different bacterial lineages was investigated using alignments of DUF164 proteins downloaded from the Sanger Institute website for PF02591 (http://pfam.sanger.ac.uk/family?acc=PF02591). Interestingly, many DUF164 proteins (43 out of 349 in the full dataset) lack one or more of the conserved cysteine residues. Most of these proteins are from bacteria belonging to either the phylum Actinobacteria or Bacteroidetes, as illustrated by Figure 2. In some cases, DUF164 proteins lack all four of the conserved cysteine residues (indicated by four stars in Figure 2). For example, DUF164 proteins from Flavobacteria (phylum Bacteroidetes) generally have the motif SXXS_X_20__ DXXS instead of the two CXXC motifs. Given the absence of the CXXC motifs in these proteins, it is unclear if they bind zinc ions or have functions similar to that of FlgZ.

**Figure 3:**
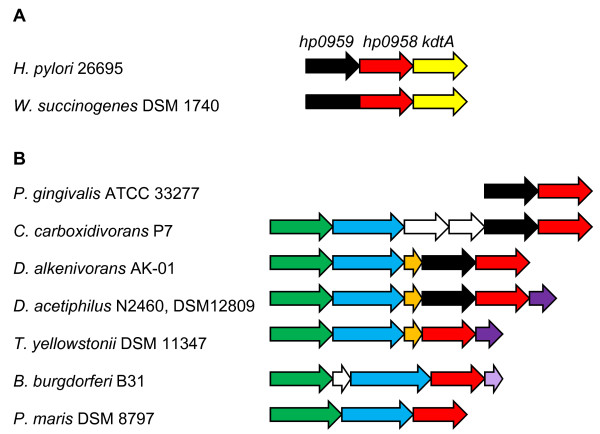
**Arrangement of genes around *flgZ *homologs in select, diverse bacteria**. (**A**) The *flgZ *gene (red) in *H. pylori *26695 is flanked by *hp0959 *(black) and *kdtA *(yellow). In *W. succinogenes *DSM 1740 the *flgZ *and *hp0959 *homologs (locus number WS2117) are fused. Arrows depicting genes are not drawn to scale. (**B**) The arrangements of genes surrounding *flgZ *homologs of bacteria from diverse taxonomic groups are indicated. Bacteria and the phylum or subphylum to which they belong (in parentheses) are: *Porphyromonas gingivalis *ATCC 33277 (Bacteroidetes); *Clostridium carboxidivorans *P7 (Firmicutes); *Desulfatibacillum alkenivorans *AK-01 (Deltaproteobacteria); *Denitrovibrio acetiphilus *N2460, DSM 12809 (Deferribacteres); *Thermodesulfovibrio yellowstonii *DSM 11347 (Nitrospirae); *Borrelia burgdorferi *B31 (Spirochaetes); and *Planctomyces maris *DSM 8797 (Planctomycetes). Genes whose products share similar functions are color coded as follows: DNA primase (*dnaG*) (green); σ^70 ^family protein (blue); tRNA-Met (orange); and RNase H (*rnhA*) (dark purple). Unique genes are indicated by white arrows, except for the gene encoding the catalytic RNA of RNase P (light purple) which is located downstream of the *flgZ *homolog in *B. burgdorferi *B31. Locus numbers for the genes (left to right) are as follows: PGN_1989 and PGN_1990 (*P. gingivalis*); CcarbDRAFT_2245 through CcarbDRAFT_2240 (*C. carboxidivorans*); Dalk_3077 through Dalk_3074 (*D. alkenivorans*); DacetDRAFT_16020 through DacetDRAFT_16060 (*D. acetiphilus*); THEYE_A1028 through THEYE_A1032 (*T. yellowstonii*); BB0710 through BB0713 plus BB_R0040 (*B. burgdorferi*); PM8707T_20723 through PM8707T_20733 (*P. maris*).

The domain organization for the 349 DUF164 proteins displayed in the Pfam database shows that the DUF164 domain is near the C-terminus in all the proteins in the database. All but three of the DUF164 proteins in the Pfam database are similar to FlgZ in that they possess a predicted coiled-coil domain near the N-terminus and lack other defined protein family domains. A notable exception to this architectural arrangement is the DUF164 protein from the Epsilonproteobacterium *Wolinella succinogenes *WS2117 which also contains a domain belonging to the NIF3 (NGG1p interacting factor 3) family (PF01784; COG0327). NIF3 interacts with the yeast transcriptional co-activator NGG1p, but the significance of this interaction is not known [37]. A gene encoding a NIF3 domain protein (*hp0959*) is located immediately upstream of *flgZ *in *H. pylori *(Figure 3A), suggesting a gene fusion event occurred in *W. succinogenes*. Gene fusion events are among the best predictors of protein-protein interactions [38]. HP0959, however, is not required for motility or RpoN stability in *H. pylori *(Figure 1A; and [19]), and we do not have any independent evidence regarding direct physical interaction between HP0959 and FlgZ. It may be that the DUF164 and NIF3 domains function together in some capacity which is unique to *W. succinogenes. W. succinogenes *appears to utilize RpoN to control expression of nitrogen-fixation (*nif*) genes in addition to flagellar genes. *W. succinogenes *also differs from *H. pylori *in that it possesses a second *rpoN *gene which appears to be part of an operon that includes several *nif *genes. It is possible that the NIF3 and DUF164 domains in *W. succinogenes *function together to modulate the activity or level of this second RpoN protein.

### Homologs of *flgZ *display shared synteny in taxonomically diverse bacteria

Since genes associated with the same cellular system or process are often co-localized in bacterial genomes, we used the JGI IMG ortholog neighborhood viewer tool to examine the gene neighborhoods surrounding *flgZ *orthologs to gain additional insight into their potential functions. As indicated previously, the gene upstream of *H. pylori flgZ *encodes a protein with a NIF3 domain. This gene arrangement is present in all of the Epsilonproteobacteria genomes in which a *flgZ *ortholog was unambiguously identified, as well as the genomes of several more distantly related bacteria, including members of Deltaproteobacteria, Bacteroidetes, Actinobacteria and several clostridia (Figure 3B). As discussed previously and shown in Figure 1A, the NIF3 domain protein is not required for motility or protection of RpoN from turnover in *H. pylori*. This does not rule out the possibility that the NIF3 domain protein functions with FlgZ in some other capacity in *H. pylori *or that the NIF3 and DUF164 domains might work together in other bacteria.

In many diverse bacterial species, genes encoding proteins containing DUF164 are closely associated with *rpoD *(encodes the primary sigma factor) and *dnaG *(encodes DNA primase) (Figure 3B). These bacteria included representative species of Spirochaetes (12 unique species), Planctomycetes (3 species), Firmicutes (7 species), Nitrospirae (1 species), Deferribacteres (1 species), and Deltaproteobacteria (8 species). The *rpoD *genes in these bacteria are located upstream of the gene encoding the DUF164 protein and may be part of the same operon. Given the close association of these genes it is possible that DUF164 proteins in these bacteria have roles in the function of the primary sigma factor.

## Conclusions

DUF164 proteins are widespread among bacteria, but the functions of these proteins are virtually unknown. To the best of our knowledge *H. pylori *FlgZ is the only member for which functions have been ascribed. Results presented here suggest the importance of the DUF164 of FlgZ in protecting RpoN from turnover. Given the co-occurrence of DUF164 and RpoN proteins in many bacterial genomes, we anticipate that DUF164 proteins have a role in RpoN function in other bacteria besides *H. pylori*. It is clear, however, that DUF164 proteins play other roles in some bacteria, such as the Actinobacteria, which lack RpoN. It is not unusual for bacteria to recruit existing genomic features to adopt additional functions, and the analysis of taxonomical distribution of FlgZ and RpoN homologs suggests that this may have been the case in the evolution of FlgZ. It is possible that the ancestral FlgZ had another function, which has been retained in some present-day bacteria, and adopted a new role in RpoN protection and flagellar regulation in some lineages. However, the decay of the CXXC motifs in some Actinobacteria and other phyla may indicate an alternative scenario where FlgZ homologs in these bacteria adopted a new function that does not require zinc coordination.

## Methods

### Bacterial strains and media

*Escherichia coli *DH5α was used for routine cloning procedures and was grown in Luria-Bertani broth or agar medium at 37^®^C. *H. pylori *ATCC 43504 and its derivatives were grown on tryptic soy agar (TSA) supplemented with 5% horse serum and grown at 37^®^C under an atmosphere of 4% oxygen, 5% carbon dioxide, and 91% nitrogen. When required, medium was supplemented with 30 μg/ml chloramphenicol or kanamycin.

### Construction and expression of FlgZ variants in *H. pylori*

Plasmid pEU39 Cm [39] is a suicide vector used to introduce DNA into the *H. pylori hp0405 *locus (encodes a NifS-like protein). The plasmid carries part of *hp0405 *that has been disrupted with a cassette containing a *Campylobacter coli *chloramphenicol transacetylase (*cat*) gene for selection of recombinants. A derivative of plasmid pEU39 Cm that carries a ~1.5-kb DNA fragment bearing *flgZ *(*hp0958*) and most of the upstream gene (*hp0959*) from *H. pylori *26695 was constructed as described previously [19]. Mutations were introduced in *flgZ *carried on this plasmid using the QuickChange II site-directed mutagenesis kit (Stratagene, La Jolla, CA) as per the supplier's instructions. Sequences for the mutagenic primers (Integrated DNA Technologies, Coralville, IA) were: 5'-CAGGCTTGTGGGGGTAGCTTTATTCGGTTGATGATAAG-3' and 5'-CTTATCATTCAACCGAATAAAGCTACCCCCACAAGCCTG-3' to change Cys-202 to serine; and 5'-CGAGTGGGGATATGATCACTAGCCCGTATTGCGGGCG-3' and 5'-CGCCCGCAATACGGGCTAGTGATCATATCCCCACTCG-3' to change Cys-223 to serine (sites where changes were introduced are underlined). The *flgZ *alleles were sequenced by the Georgia Genomics Facility at the University of Georgia to confirm that the correct mutations had been introduced and that no other mutations had been introduced inadvertently. Plasmids bearing the mutant *flgZ *alleles were introduced by natural transformation into a *H. pylori *ATCC 43504 containing a *flgZ*:*aphA3 *mutation [19]. Recombinants were selected on TSA supplemented with serum and chloramphenicol and the presence of the *flgZ *alleles in the *hp0405 *locus was confirmed by PCR.

### Motility assays

Motility agar plates consisted of Mueller-Hinton broth supplemented with 5% horse serum and 0.35% agar. Sterile toothpicks were used to inoculate motility agar with *H. pylori *strains. Motility of *H. pylori *strains was scored after incubating plates at 37^®^C under an atmosphere of 4% oxygen, 5% carbon dioxide, and 91% nitrogen for 4 to 5 days.

### Western blot analysis

Immunoblotting with primary antibodies directed against maltose binding protein (MBP) fusions to FlgZ, RpoN or FlaB was done as described previously [19,29]. Antibodies directed against MBP-RpoN were affinity purified prior to use as described [19]. Primary antibodies bound to proteins immobilized on the membranes were detected by enhanced chemiluminescence using peroxidase-coupled goat anti-rabbit antibody (MP Biomedicals, Aurora, ID).

### Analysis of genome sequences

FlgZ and RpoN homologs were identified from the United States Department of Energy's JGI IMG and Wellcome Trust Sanger Institute Pfam databases [40,41]. Genes surrounding *flgZ *homologs were analyzed using the JGI ortholog neighborhood viewer tool. The taxonomical distribution of FlgZ and RpoN homologs was compiled from data downloaded from the IMG database using an in-house computer program.

## List of abbreviations

FlgZ: flagellar-associated zinc-ribbon protein; NIF3: NGG1p interacting factor 3; JGI: Joint Genome Institute; IMG: Integrated Microbial Genomes; TSA: trypic soy agar; cat: chloramphenicol transacetylase; MBP: maltose-binding protein; aphA3: kanamycin-resistance gene

## Competing interests

The authors declare that they have no competing interests.

## Authors' contributions

LP constructed and characterized the *H. pylori *strains used in the study. JT participated in the taxonomic analysis. JM participated in the taxonomic analysis, performed the statistical analysis of the data, and helped draft the manuscript. TH conceived of the study, participated in its design, and drafted the manuscript. All authors read and approved the final manuscript.
